# Transformation of ZnO polycrystalline sheets into hexagon-like mesocrystalline ZnO rods (tubes) under ultrasonic vibration

**DOI:** 10.1186/1556-276X-9-214

**Published:** 2014-05-07

**Authors:** Jianning Ding, Xiang Fang, Rong Yang, Biao Kan, Xiazhang Li, Ningyi Yuan

**Affiliations:** 1 Jiangsu Collaborative Innovation Center of Photovolatic Science and Engineering, Changzhou University, Changzhou, Jiangsu 213164, China; 2 Jiangsu Key Laboratory for Solar Cell Materials and Technology, Changzhou, Jiangsu 213164, China; 3 Center for Low-Dimensional Materials, Micro-Nano Devices and System, Changzhou University, Changzhou, Jiangsu 213164, China

**Keywords:** ZnO, Mesocrystalline, Polycrystalline sheet, Hexagonal-like rod, Hexagonal-like tube

## Abstract

The mesoscale assembly process is sensitive to additives that can modify the interactions of the crystal nucleus and the developing crystals with solid surfaces and soluble molecules. However, the presence of additives is not a prerequisite for the mesoscale transformation process. In this study, ZnO sheet networks were synthesized on Al foils by a hydrothermal process. Scanning electron microscopy and transmission electron microscopy images confirmed that under ultrasonic vibration, monolithic polycrystalline ZnO sheets transformed into hexagon-like mesocrystalline tubes or rods. The formation mechanism was discussed.

## Background

Zinc oxide (ZnO), a wide-band gap II-VI semiconductor, has a wurtzite structure, belongs to the space group *C*6*mc*, and has lattice parameters of *a* = 0.3249 nm and *c* = 0.5207 nm [1]. The wurtzite structure of ZnO can be described as a number of alternating planes composed of tetrahedrally coordinated O^2−^ and Zn^2+^ ions stacked along the *c*-axis. The oppositely charged ions produce positively charged Zn (0001) and negatively charged O polar surfaces [1]. Together with the polar surfaces, three fast growth directions along [0001], $\left[10\bar{1}0\right]$, and $\left[2\bar{1}\bar{1}0\right]$ facilitated anisotropic growth of the one-dimensional (1D) ZnO structures, including *c*-axis-oriented nanowires and *a*-axis-oriented nanobelts [2-5].

Recently, a new class of nanostructured solid materials, mesocrystals, consisting of self-assembled crystallographically oriented nanoparticles [6-8] has attracted much attention. A large variety of ZnO mesocrystals grown using different additives has been obtained [9-14]. During the crystal growth of mesocrystals, the primary particles involved are usually scattered in the solution and are formed through the spontaneous organization to produce crystallographically continuous particles and ordered structures. For example, hexagonal, nanoplatelet-based, mesocrystalline ZnO microspheres were grown using a facile solution-based route [15]. Several mechanisms of mesocrystal formation have been proposed: biomineralization, roles of organic additives, alignment by capillary forces, hydrophobic forces, a mechanical stress field, magnetic fields, dipole and polarization forces, external electric fields, minimization of the interfacial energy, and so on [16-23]. However, the mechanisms are, however, still under debate.

In this work, ZnO polycrystalline sheets were synthesized on Al foils by a hydrothermal process. It is very interesting to find that the monolithic polycrystalline sheets could be transformed into hexagon-like mesocrystalline tubes or rods under ultrasonic vibration. To the best of our knowledge, this is the first report of such a transformation.

## Methods

ZnO sheet networks were synthesized on Al foils by a hydrothermal process. Previous to growing, the Al foil surface was processed with ultrasonic cleaning in acetone, alcohol, and deionized water for 20 min, respectively. The hydrothermal growth was carried out by immersing the Al foils in an aqueous solution containing zinc nitrate hexahydrate (Zn(NO_3_)_2_ · 6H_2_O, 10 mM) and methenamine ((CH_2_)_6_ N_4,_ also called hexamethylenetetramine or HMT, 10 mM) at 90°C in a stainless steel autoclave for 2 h. After cooling to room temperature naturally, the ZnO-coated Al foils were first washed with water and then ethanol to remove the organic residues. The foils were then baked at 70°C for 1 h to obtain dried ZnO-coated Al foils. An X-ray diffractometer with Cu *K*_*α*_ radiation (D/max 2500 PC, Rigaku Corporation, Shibuya-ku, Japan, 2*θ*/*θ*, = 0.1542 nm) at 40 kV was used to analyze the crystalline structures of the as-grown ZnO on Al foils.

The dried ZnO-coated Al foils were placed in ethanol for exposure to ultrasonic vibration at 0°C for 20 to 50 min to observe the morphological transformation of the ZnO on the Al foils. Besides, the ZnO nanosheets on Al substrate were scraped off from the substrate and were added into ethanol to be dispersed by ultrasonication for 0.5 h. The dispersed ZnO samples are also investigated. Field-emission scanning electron microscope (FESEM, SUPRA55, German) images were obtained and recorded on a LEO 1530 VP, with the voltage of 5 kV and spot size of 20 mm. Transmission electron microscope (TEM, JEOL JEM-2100,200 kV, Akishima-shi, Japan) images were observed on a JEM 200CX to further investigate the morphological and structural transformation of ZnO.

## Results and discussion

**Figure 1:**
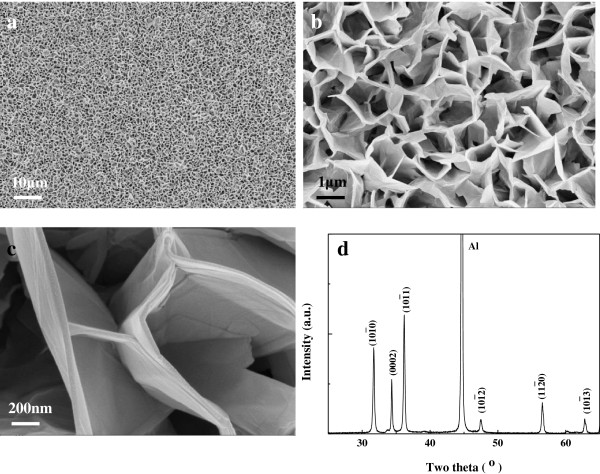
SEM images of ZnO sheets grown on Al foils (a, b, c) and XRD data of ZnO sheet (d).

Figure 1a,b,c shows FESEM images of the ZnO grown on the Al foils, which are similar to the previously reported results [24]. For the sample grown at 90°C for 2 h, the low-magnification image in Figure 1a indicates that the ZnO sample had good uniformity on a large scale, displaying sheet-like morphologies, with the sheets displaying random orientations. From the high-magnification image shown in Figure 1b, we can see that the ZnO sheets were connected to each other and formed networks. The average dimensions of the observed sheets were in the range of 2 to 3 μm with a thickness of 20 to 30 nm. Figure 1c shows that these nanosheets exhibited a curved morphology with a smooth surface.

The crystallinity of the as-grown products on Al foils were examined using X-ray diffraction (XRD). Figure 1d shows the XRD pattern for the ZnO nanosheet. All the indexed peaks in the spectrum were well matched with the hexagonal wurtzite phase of bulk ZnO. With the exception of the peak appearing at 44.7° corresponding to Al foil, the other peaks appearing at 31.7°, 34.4°, 36.3°, 47.5°, 56.5°, and 62.9° corresponded to the $\left(10\bar{1}0\right)$, (0002), $\left(10\bar{1}1\right)$, $\left(10\bar{1}2\right)$, $\left(11\bar{2}0\right)$, and $\left(10\bar{1}3\right)$ planes of ZnO, respectively, indicating that the only product obtained was wurtzite ZnO. The formation of ZnO nanosheets could be attributed to the Al substrate. HMT acted as a weak base that slowly hydrolyzed in the solution with water and gradually produced OH^−^, while zinc ions were released by Zn(NO3)_2_. The ZnO growth process is shown by the chemical reactions listed as follows:

(1) $${\left({\mathrm{CH}}_{2}\right)}_{6}N_{4}+6H_{2}O\to 4{\mathrm{NH}}_{3}+6\mathrm{HCHO}$$

(2) $${\mathrm{NH}}_{3}+H_{2}O\to {{\mathrm{NH}}_{4}}^{+}+{\mathrm{OH}}^{-}$$

(3) $${\mathrm{Zn}}^{2+}+2{\mathrm{OH}}^{-}\to \mathrm{Zn}{\left(\mathrm{OH}\right)}_{2}$$

(4) $$\mathrm{Zn}{\left(\mathrm{OH}\right)}_{2}\to \mathrm{ZnO}+H_{2}O$$

It is well known that the fastest growth rate of ZnO is along the [0001] direction owing to the lowest surface energy of the (0002) facet under thermodynamic equilibrium conditions, resulting in the growth of ZnO nanorods on most occasions. However, when Al was used as a substrate in our study, it absorbed OH^−^ ions to form Al(OH)_4_^−^ on the surface, which adhered to the Zn^2+^-terminated (0001) surface and suppressed growth along the [0001] direction, resulting in lateral growth of ZnO [25,26]. Meanwhile, the precipitation of aluminum hydroxide (Al(OH)_3_) also reduced OH^−^ concentration, supersaturating the growth solution. Owing to the influence of Al foils, 1D nanorods with the *c*-axis along the [0001] direction were not formed. In contrast, two-dimensional (2D) ZnO sheets were formed, which exhibited crooked nanoplate morphology instead of a freely stretched shape, suggesting that there was stress in the ZnO sheets.

**Figure 2:**
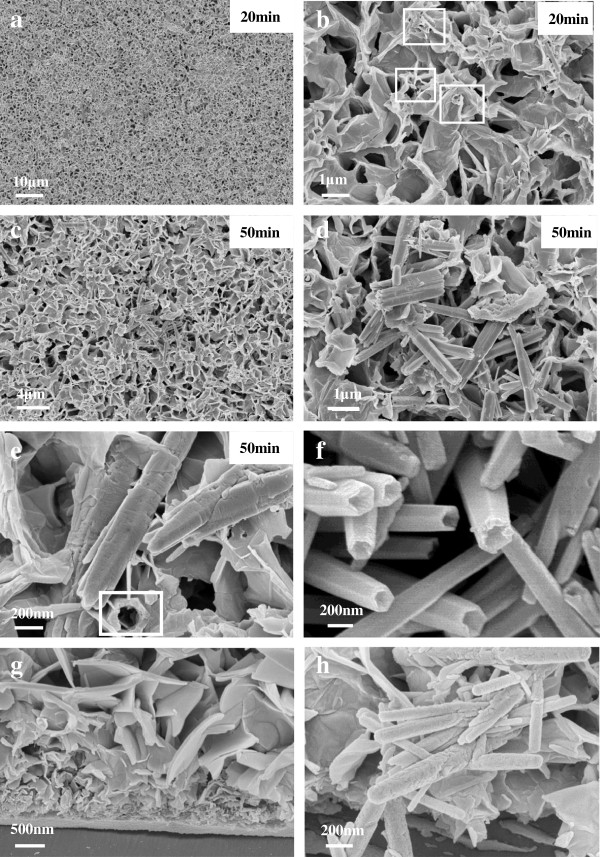
**ZnO sheet networks formed on an Al foil upon ultrasonication.** Low **(a)**, high **(b)** magnification SEM images of ZnO on Al foils after 20 min ultrasonication vibration, **(c, d, e)** SEM images of ZnO on Al foils after 50-min ultrasonication vibration, **(f)** SEM images of ZnO on Al foils after 50 min ultrasonication vibration, **(g, h)** cross-sectional SEM images of the sample before and after ultrasonic treatment.

Figure 2 shows the ZnO sheet networks formed on an Al foil upon ultrasonication. As shown in Figure 2a, the ZnO sheet networks were destroyed after 20 min of ultrasonication and some sheets wrinkled. The high-magnification SEM images revealed more that some sheets began to curl (indicated by squares in Figure 2b). With the vibration time extended to 50 min, 1D ZnO nanostructures including nanorods and nanotubes were observed, as shown in Figure 2c,d,e. Because the ZnO sheets were connected to each other, many remained connected when they transformed into 1D structures. Regardless of whether they were connected, it should be noted that the nanorods or nanotubes formed from the original ZnO sheets exhibited hexagon-like structures. The diameter and length of the formed nanorods or nanotubes were around 200 to 300 nm and 2 to 3 μm, respectively, while the thickness of the nanotube walls was around 70 to 80 nm (as indicated by the square in Figure 2e). Figure 2f is the SEM image taken from the ZnO sample scraped off from the Al substrate and then added into ethanol to be dispersed by ultrasonication for 0.5 h. It is observed that all the original ZnO nanosheets have turned into hexagon-like nanotubes. It is believed that these 1D structures were formed by layer-by-layer winding of the nanosheets. In order to prove that the nanorods/tubes are formed during the ultrasonic process but not generated in the hydrothermal process that may be covered by nanosheets, the ZnO nanosheet-covered Al foil was bended and placed into the ultrasonic wave. Figure 2g,h showed the cross-sectional SEM images of the sample before and after ultrasonic treatment. Apparently, some layers of tiny nanosheets are stacked on the surface of substrate at the earlier stage of hydrothermal process, after which ZnO nanosheets with larger sizes were synthesized continuously. It is important to note that there are no nanorods or nanotubes hidden in the nanosheets. However, after the ultrasonic process, large numbers of nanorods or nanotubes appeared, as shown in Figure 2h. The results were consistent with the above description and confirmed the claim further.

**Figure 3:**
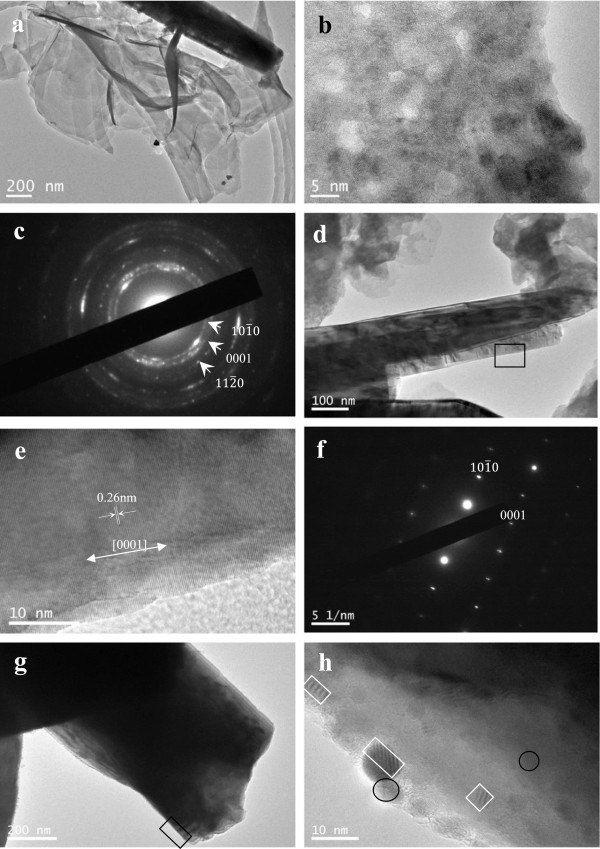
**TEM images and SAED patterns. (a, b)** TEM images of ZnO nanosheet, **(c)** selected area electron diffraction (SAED) pattern of nanosheet, **(d, e, g, h)** TEM images of nanorod, **(f)** SAED pattern of nanorod.

Further structural characterization of ZnO was performed by TEM, high-resolution TEM (HRTEM), and selected area electron diffraction (SAED). Figure 3a shows a TEM image of some stacking ZnO nanosheets with a nanorod lying alongside. Figure 3b depicts a typical HRTEM image of a nanosheet, where it was found that the crystal consisted of ZnO polycrystalline grains. The SAED pattern (Figure 3c) showing diffused rings and regular spots also confirmed the above result. Figure 3e shows an HRTEM image taken from the part of the rolled-up nanorod (marked by the box in Figure 3d. The clear fringes correspond to the (0002) plane of hexagonal ZnO, indicating that [0001] was the longitudinal direction for the formed ZnO nanorods or nanotubes. The sharp and bright dots in the SAED pattern (Figure 3f) indicate that the nanorod was single-crystalline-like structure. The SAED and HRTEM results both demonstrated the single-crystalline-like feature of the ZnO nanorods. However, we also discovered many defects in some nanorods (Figure 3g) transformed from nanosheets. Figure 3h is an HRTEM image taken from the part of the rolled-up nanorod (marked by the box in Figure 3g). Some clear moiré patterns appear in the square box in Figure 3h, which were created when two repetitive patterns (two sets of parallel lines in the current case) overlapped at a very small angle. This indicated that the ZnO nanorods were indeed mesocrystals built from thin nanosheets. Besides, there were some nanocrystals (shown in the circle in Figure 3h) with orientations that were not completely aligned. Together, the moiré patterns and the unaligned nanocrystals confirmed that the mesocrystalline nanorods or nanotubes were transformed from polycrystalline ZnO nanosheets.

It was suggested that the nanosheet rolled up along the [0001] direction primarily as a result of the minimization of the surface energy. As shown in Figure 1b,c, the interlinked ZnO nanosheets were in crooked rather than freely stretched shapes, which indicated that there existed stress in ZnO nanosheets. When the ZnO nanosheets were separated from the substrates under ultrasound vibration, the stress would be released. And the nanosheets would begin to wind around each other layer by layer, and the short-range chemical bonds among these layers resulted in nanorods or nanotubes. The reduced surface area and the formation of chemical bonds (short-range forces) between the layers should be responsible for stabilizing the coiled structure. As for the formation of mesocrystalline ZnO rods (tubes) rather than polycrystalline ones, the dipole-dipole interaction was considered the driving force [27-30]. For the polycrystalline ZnO sheets, the measured interplanar distances of most single-crystalline nanosize grains are 0.265 nm, corresponding (0001) axis of ZnO. Along (0001) axis, the oppositely charged ions produce positively charged Zn (0001) and negatively charged O $\left(000\bar{1}\right)$, which forms a dipole. Under ultrasonic vibration, these dipoles were aligned by the dipole-dipole interaction, and the mesocrystalline ZnO rods were formed. The dipole-dipole interaction has been suggested as the mechanism of mesocrystal formation [31-33]. Differently, in our work, the nanocrystals were not dispersed in the organic solvent. The hexagon-like external morphology of mesocrystal ZnO rods or tubes were thought to be determined by hexagonal wurtzite structure of ZnO.

## Conclusion

ZnO nanosheets with a large area and a small thickness were prepared on Al substrates. Under ultrasonic vibration, these monolithic polycrystal ZnO nanosheets rolled up and transformed into mesocrystalline nanorods or nanotubes. It was suggested that the transformation of nanorods or nanotubes from nanosheet primarily as a result of the minimization of the surface energy. The mesocrystal formation was thought ascribed to the dipole-dipole interaction.

## Competing interests

The authors declare that they have no competing interests.

## Authors' contributions

JD and NY defined the research theme and designed the experiments. XF and RY carried out the studies, participated in the sequence alignment, and performed the statistical analysis. JD, NY and XF drafted the manuscript. BK conceived of the study and participated in its design. XL participated in analysis of data and coordination. All authors read and approved the final manuscript.
